# Singing for memory: neural and cognitive effects of a choral intervention in older adults

**DOI:** 10.3389/fnagi.2025.1679873

**Published:** 2025-11-19

**Authors:** Miriam Napadow, Håkan Fischer, Maria Sandgren, Máté Magyar, Zsuzsanna Lénárd, László Harmat, Örjan de Manzano

**Affiliations:** 1 Department of Psychology, Linnaeus University, Växjö, Sweden; 2 Department of Neurobiology, Care Sciences and Society, Aging Research Center, Karolinska Institutet, Stockholm, Sweden; 3 Stockholm University Brain Imaging Centre, Stockholm, Sweden; 4 Department of Psychology, Stockholms Universitet, Stockholm, Sweden; 5 Department of Behavioural Sciences and Learning (IBL), Linköping University, Linköping, Sweden; 6 Mälardalens Universitet, School of Health, Care and Social Welfare, Västerås, Sweden; 7 Department of Neuroradiology, Medical Imaging Centre, Faculty of Medicine, Semmelweis Egyetem, Budapest, Hungary; 8 Department of Radiology, Medical Imaging Centre, Faculty of Medicine, Semmelweis University, Budapest, Hungary, Budapest, Hungary; 9 Max Planck Institute for Empirical Aesthetics, Frankfurt am Main, Germany; 10 Department of Neuroscience, Karolinska Institutet, Stockholm, Sweden

**Keywords:** choir singing, cognitive reserve, episodic memory, healthy aging, neuroimaging

## Abstract

**Introduction:**

Lifestyle factors are important predictors of successful aging, and targeted interventions could be key to mitigating the negative effects of aging. Episodic memory is of particular interest as it is notably sensitive to aging. Given the social, intellectual, and physical stimulation that choral singing provides, along with the enjoyment it offers which is a strong motivator, it has been suggested as a particularly promising intervention to promote successful aging.

**Method:**

Thirty-four participants, aged 65 to 75 at recruitment, took part in a choral singing intervention involving 47 weekly 1.5-h rehearsals. The study included examinations at three time points: T1, T2, and T3. A control period (T1-T2) was followed by the intervention period (T2-T3), each lasting approximately 11 months. At each assessment, episodic memory was measured with the Wechsler Memory Scale (WMS-LMI, WMS-LMII), and participants completed an fMRI Face-Name Paired Associates Task (FN-PA) to examine brain activity during memory encoding and retrieval.

**Results:**

Partial correlation analyses, adjusting for age and cognitive ability, showed significant improvements in episodic memory following both the control period (T1-T2) and the choir intervention (T2-T3), but only the latter scaled with rehearsal attendance. Right hippocampal activity during encoding in the FN-PA task also correlated with attendance, and with age. Additionally, task-dependent functional connectivity increased between the right lateral prefrontal cortex, left posterior fusiform cortex and left hippocampus, while connectivity between the right lateral prefrontal cortex and the left inferior frontal gyrus decreased after the intervention.

**Discussion:**

These findings suggest that regular participation in choral singing may enhance episodic memory and have a positive influence on related brain networks in older adults. The suggestive dose–response effect highlights choir singing as an engaging, multifaceted activity with the potential to contribute to cognitive resilience in aging populations.

## 1 Introduction

There are large individual differences in successful aging (Josefsson et al., 2012), and lifestyle has been shown to be an important factor when it comes to maintaining healthy well-being and cognition later in life (Andel et al., 2016). Given the increasing proportion of elderly in our society, a key issue is to explore ways to promote healthy aging and identify strategies that help older adults maintain their abilities and functions. It has been suggested that targeted interventions, which are both physically and intellectually engaging, may help maintain cognitive health in older adults (Håkansson et al., 2016; Kivipelto and Håkansson, 2017; Nyberg et al., 2020). Cultural and musical activities have also been suggested as potential interventions (Alain et al., 2014). However, research on how musical activities might have a beneficial influence on cognition, health, and well-being among older adults is still very limited.

Choral singing is one of those activities that combines several elements known to promote health: intellectual (Daffner, 2010) and physical challenge (McPhee et al., 2016), social stimulation (Douglas et al., 2016), and the added benefit of being an enjoyable experience (Moss et al., 2018) that evokes a sense of meaning (Rickard McCoy, 2016). There are numerous group activities that promote social interaction—such as sports, study circles, and hobby groups—but choral singing possesses a unique combination of qualities worth highlighting. First, it is an activity where the entire group works towards a common goal, requiring synchronization in tempo and harmony, which can facilitate experiences of group cohesion (Stewart and Lonsdale, 2016; Bonshor, 2013). Second, choral singing is highly accessible; no prior formal musical training is necessary, and participants can experience the joy of “making music” from the very first session. Third, choral singing remains feasible for people with age-related physical limitations. Finally, choral singing is both a non-pharmaceutical and a cost-efficient activity that promotes both mental and social well-being.

In the present study, we aim to examine the impact of a choral singing intervention on adult aging, focusing on neural processes and cognitive health markers during the performance of an fMRI episodic memory task. Thus far, the field of neuroimaging only minimally explored the effects of choral singing on the brain, and its impact on older adults remains even more understudied (Tan et al., 2018). While previous research has shown that musical training is associated with cognitive benefits both in childhood and adolescence (Wolff et al., 2023), as well as during middle adult life (Vetere et al., 2024), few studies have investigated whether older adult beginners might also experience similar advantages. The present study aims to address both these gaps.

### 1.1 Age-related changes in episodic memory

Episodic memory, that is, the type of declarative memory that stores events linked to personal experiences, is the most age-sensitive of the memory functions (Nyberg and Pudas, 2019). The onset of changes in adult aging usually starts from the age of 60 and onwards. Nevertheless, there are large variations in individual trajectories (Nyberg, 2017). Furthermore, recall and recognition of episodic events appear to be affected differently in normal aging: recall tends to weaken, while recognition remains relatively intact (Nyberg, 2017). A frequently discussed concept in aging research is ‘cognitive reserve’, which refers to the ability to resist cognitive decline and maintain resilience. Previous research suggests that cognitive reserve scales with education level and intelligence (Schmand et al., 1997; Boyle et al., 2021). Pudas et al. (2014) found that individual differences in cognitive ability during midlife can predict brain activation patterns during episodic memory tasks in older age. Interestingly however, some studies also suggest that social and physical activities may function as protective factors against episodic memory decline (Josefsson et al., 2012).

### 1.2 Brain changes in aging

The neural correlates of cognitive aging are complex and multifactorial, involving a combination of structural and functional changes in the brain (Kalpouzos and Persson, 2025). Aging is typically accompanied by grey matter atrophy, decreased white matter integrity, and altered brain connectivity, which may be linked to declines in cognitive functioning (Madden et al., 2009; Good et al., 2001). Previous research has for instance shown that hippocampal shrinkage is a key feature of aging and contributes to deficits in episodic memory (Raz and Rodrigue, 2006). The prefrontal cortex (PFC), responsible for higher-order functions such as decision-making, planning, and self-regulation, also undergoes age-related changes (Raz et al., 2005). Beyond structural changes, functional brain activity also shifts with age.

Although age-related changes occur, there is evidence of the brain’s remarkable ability to adapt through neuroplasticity, and compensatory mechanisms may help older adults maintain performance despite structural decline (Grady, 2012). The posterior–anterior shift in aging (PASA) is a well-documented pattern of brain reorganization, presented by Davis et al. (2008), and frequently observed in functional neuroimaging studies of aging (Kalpouzos and Persson, 2025). It reflects an age-related increase in frontal brain activity coupled with reduced activation in posterior regions, particularly during cognitively demanding tasks. PASA is commonly interpreted as a compensatory mechanism for age-related cognitive decline, where frontal regions are recruited more heavily to offset declines in posterior sensory regions, and most results suggest a positive association between this reorganization and performance. PASA was originally observed during working memory tasks but has also been observed during other tasks, for instance episodic memory tasks; it has therefore been argued that PASA reflects a general compensatory mechanism across multiple cognitive domains (e.g., Davis et al., 2008). Findings are somewhat mixed however (Johansson et al., 2020; Morcom and Johnson, 2015), and it remains unclear under what circumstances the reorganization should be interpreted as beneficial. For instance, Morcom and Henson (2018) challenged the compensatory interpretation of PASA, suggesting that increased frontal activity in older adults might not always be beneficial and could reflect less efficient processing rather than compensation. Similarly, Ansado et al. (2013), in their review, suggested that PASA might not universally represent a compensatory mechanism and could vary depending on the cognitive task and individual differences among older adults.

Episodic memory tasks are especially interesting because they involve both posterior sensory regions (e.g., for stimulus encoding) and frontal control regions (e.g., for strategy use, retrieval monitoring). There has been limited discussion of PASA in the context of interventions or how activation patterns might change in response to targeted treatments such as cognitive training, physical exercise, or pharmacological treatments. Most existing work has relied on cross-sectional comparisons (young vs. older adults) or longitudinal studies without experimental manipulation. This is notable, because Nyberg et al. (2010) found that longitudinal and cross-sectional analyses demonstrated opposite effects for a semantic categorization task performed by older adults; cross-sectional analyses indicated over-recruitment of frontal regions with increasing age (in line with PASA), while the longitudinal analyses rather showed under-recruitment. It is unclear whether the same pattern of results would be exhibited also in episodic memory tasks.

With regard to episodic memory, both the PFC and hippocampus are consistently implicated (Nyberg, 2017; Eichenbaum, 2017). Studies have also shown that high performing older adults engage these regions to a greater extent during episodic memory tasks compared to average performers (Pudas et al., 2013). Furthermore, successful older memory performers exhibit lower regional spontaneous brain activity in hippocampus during rest (Bråthen et al., 2018), and higher functional connectivity between the hippocampus and the default mode network, possibly reflecting more efficient network regulation. In contrast, typical older adults showed reduced activity in the left posterior cingulate cortex, left superior frontal cortex, as well as the left fusiform area, compared to younger adults (Uslu et al., 2023) when performing a face recognition task.

Another key aspect of functional aging is the distinction between encoding and retrieval processes. Salami et al. (2012) identified both a general encoding-retrieval network, activating the frontoparietal cortex and posterior hippocampus, and task-specific activations. For example, the anterior hippocampus was less engaged during encoding. These findings support the HERA (Hemispheric Encoding/Retrieval Asymmetry) model by Tulving et al. (1994), which posits that encoding is typically associated with the left PFC, while retrieval engages the right PFC in younger adults. Aging is associated with a reduction in this hemispheric asymmetry, as described by the HAROLD (Hemispheric Asymmetry Reduction in Older Adults) model (Cabeza, 2002). This process has been associated with impaired episodic memory (Johansson et al., 2020). It is unclear whether bilateral activation reflects either a compensatory strategy, or a dedifferentiation process, where specialized neural systems become less distinct with age (Reuter-Lorenz et al., 2000).

### 1.3 Choral singing, memory and fMRI studies

During the past decade, there has been a steady increase in research assessing the potential benefits of musical activities improving various aspects of life, including cognitive reserve in older adults (for a review, see Roman-Caballero et al., 2018). Previous research indicates that a short-term musical intervention, such as piano playing or rhythmic training with percussive instruments, can improve cognitive functions in older adults (for a review, see Ferreri et al., 2019). A more recent review (Espinosa et al., 2025) acknowledges that few studies have assessed the neurobiological effects of music-making interventions for older adults, and even fewer have focused on choral singing. Furthermore, it underlines the lack of functional studies. Singing is a multifaceted activity that simultaneously engages auditory, motor, linguistic, cognitive, and emotional systems in the brain (reviewed in Tragantzopoulou and Giannouli, 2025). This neural complexity is one of the reasons singing offers such an interesting model for exploring the relation between cognitive activity and cognitive reserve.

There have been mixed reports regarding differences in connectivity between singers and non-singers. Zamorano et al. (2023) conducted a resting-state fMRI study and found that trained singers, compared to non-singers, exhibited enhanced connectivity between the insula—a region involved in many cognitive functions, including attention and emotional processing—and the bilateral superior parietal lobes and cerebellum, which are involved in various sensorimotor processes. In contrast, Zhang and Tremblay (2023) examined amateur singers and non-singers, but found no significant differences in resting-state connectivity between the auditory, speech, language, or dorsal attention networks. They did however observe that connectivity between these networks decreased significantly with age in both groups.

Pentikäinen et al. (2022) using EEG found that choral singing had a positive impact on complex auditory encoding in healthy adults. In a separate study, Pentikäinen et al. (2022) also found that choral singers demonstrated higher verbal flexibility and better social integration compared to non-singers. In a 2-year follow-up study (Pentikäinen et al., 2023) of the same sample, however, singers showed a stable performance on the verbal flexibility task, while the non-singers improved.

To our knowledge, no fMRI studies have specifically explored the relationship between aging, episodic memory, and participation in choral singing. While music interventions have been shown to improve memory and executive function in clinical populations (e.g., Rajakumar and Mohan, 2024), research on the effects of choral singing on memory in non-clinical groups, especially using neuroimaging, remains scarce. A few studies, however, are worth noting. For instance, Feng et al. (2020) conducted a randomized controlled trial with participants at risk for dementia. One group engaged in regular choral singing for two years, while the other group received health education. While no significant structural brain changes were observed, the choir singers scored significantly better on a composite cognitive test following the intervention. Another study by Perron et al. (2022) found that age-related speech-perception-in-noise difficulties, through plasticity in the auditory cortical and dorsal speech regions, could be reduced by choral singing. In addition, they found that practice behavior, for example home practice, singing in several languages, and formal singing training, were important factors for improvements.

Lastly, several researchers (Moisseinen et al., 2025; De Godoy et al., 2021; Damoiseaux, 2017; Tragantzopoulou and Giannouli, 2025) have recommended conducting more longitudinal studies in the fields of singing, neuroscience, and aging. Their main reasons have been to address the complexity of cognitive aging and to be able to compare the results of brain changes related to aging with findings from other studies. Furthermore, it has been proposed that longitudinal designs could improve understanding of long-term effects and generalizability.

### 1.4 Research questions and hypotheses

The present study was based on an intervention in the form of an 11-month choral singing course specifically designed for healthy participants between the ages 65–75 years. Participants performed an fMRI experiment with an episodic memory task (Face-Name Paired Associate learning task, or FN-PA), as well as a session of psychological testing at three timepoints approximately 11 months apart. The time between the first and second timepoints constituted a passive control period; the choral singing intervention was carried out between the second and third timepoints. The aim of the current study was to investigate whether the choral singing intervention would benefit episodic memory; and if so, explore potential changes in neural activity during the FN-PA task post- vs. pre intervention to learn more about functional plasticity related to cognitive reserve.

The within-subjects design is preferable in this study for several reasons. First, it controls for large inter-individual variability in episodic memory and neural activity due to factors like age, education, and health, increasing statistical power by comparing each participant to themselves. Second, it requires fewer participants than a between-subjects design, which is advantageous in resource-intensive fMRI research. Third, it is ethically favorable, as all participants receive the potentially beneficial choral singing intervention, avoiding the issue of withholding it from a control group. Fourth, the inclusion of three timepoints—before, during a passive control period, and after the intervention—allows the researchers to distinguish natural aging, learning and habituation effects from intervention-specific effects. Finally, since the aim is to examine changes in neural activation related to functional plasticity and cognitive reserve, tracking within-person changes provides more precise insight than group comparisons alone.

Building on models of age-related neural change, several special hypotheses could be proposed regarding the effects of the choir intervention. According to the PASA model, choir practice may enhance processing efficiency in posterior regions, thereby reducing the need for compensatory frontal over-recruitment. In other words, if posterior memory systems become more effective, reliance on frontal compensation might decrease. The HAROLD model would predict a restoration of hemispheric specialization, particularly in the context of episodic memory, aligning with the HERA model’s proposal of left-lateralized encoding and right-lateralized retrieval. Similarly, the CRUNCH (Reuter-Lorenz and Mikels, 2006) model suggests that the intervention could result in more efficient, selective recruitment of task-relevant regions (such as the hippocampus and posterior regions), reducing unnecessary frontal activation at lower task demands. With regard to functional connectivity, we might expect enhanced integration between memory-related regions—especially between the hippocampus and areas such as the fusiform gyrus, given the role of facial stimuli in our experimental task.

## 2 Materials and methods

### 2.1 Participants

The participants were recruited through advertisements on social media and in local newspapers (Stockholm area, Sweden). The inclusion criteria were the following; healthy adults aged 65–75 years at the time of recruitment (fall/winter 2019–2020), normal vision or normal when corrected, at least 7 years of education, scoring at least 26 points on Mini-Mental State Examination (MMSE) (Folstein et al., 1975), scoring within 2 SD of the age-appropriate mean on the Wechsler Memory Scale Logical Memory I (WMS-LMI) and Logical Memory II (WMS-LMII) (Wechsler, 1997), and scoring below 10 points on the Geriatric Depression Scale (GDS) (Yesavage et al., 1982).

The exclusion criteria were; participation in choir signing during the last 10 years, having previous or existing medical conditions involving or affecting on the central nervous system based on DSM-5 criteria (American Psychiatric Association, 2013), self-reported mental illness, and hearing loss that would directly affect participation in choir singing activities. In addition, individuals with contraindications to brain imaging with MRI, such as having a pacemaker, metal implants, or claustrophobia, were excluded.

**Figure 1:**
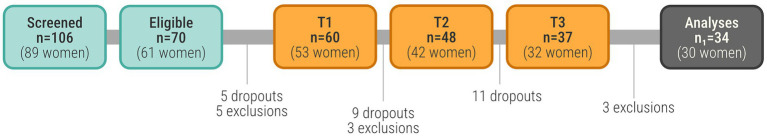
The number of participants at each stage of the data collection.

In total, 106 (89 women) participants were considered and screened for the study (see Figure 1) out of which 70 (61 women) met the inclusion criteria and were scheduled for the first data collection (T1). Before completing T1, 5 participants dropped out and another 5 were excluded due to: scoring below cutoff on WMS-LMI (3 participants); self-reported mental illness (2 participants). After T1, 60 participants remained that had both completed all tests at T1 and met the inclusion criteria. Before T2, 9 participants dropped out and three were excluded due to: an assessment from the radiologist, and technical issues with scanner at time of scanning (2 participants) that was discovered. After T3, 37 participants remained in the sample. Another three participants were excluded from the MR-analyses due to; not successfully completing the Raven’s test (2 participants), scoring below 26 points on the WMS-LMI (1 participant). In the final sample, 34 participants remained for analyses. We conducted an *a priori* power analysis using G*Power for a repeated-measures ANOVA (within-subjects, 3 time points), assuming a medium effect size (*f* = 0.25), *α* = 0.05, and power (1–*β*) = 0.80. This analysis indicated that a minimum of 28 participants would be required to detect the intended effects. Our final sample of 34 completers therefore exceeded this threshold, suggesting that the study was adequately powered for the primary analyses.

Informed, written consent was obtained by all participants, and the study was approved by the Swedish Ethical Review Authority (Dnr: 2023-02310-02). Each participant received a cinema ticket as compensation after each data collection (i.e., maximum 3 in total).

### 2.2 Choir intervention and study design

The choral singing intervention course was a product of a mixed-method study with the purpose of gaining actionable knowledge on how to design the choir intervention for a positive experience in the target group (beginners aged 65–75) and cultural context (Stockholm area) (Napadow et al., 2024). Examples of topics studied was participants’ preferences of competence and leadership qualities of the choir conductor, practices of rehearsal and concerts, issues related to sense of meaning and coherence, as well preferences for rehearsal room features (acoustics, equipment, etc.). Below is a description of the choir intervention design; a more detailed description can be found in Napadow et al. (2024).

**Figure 2:**
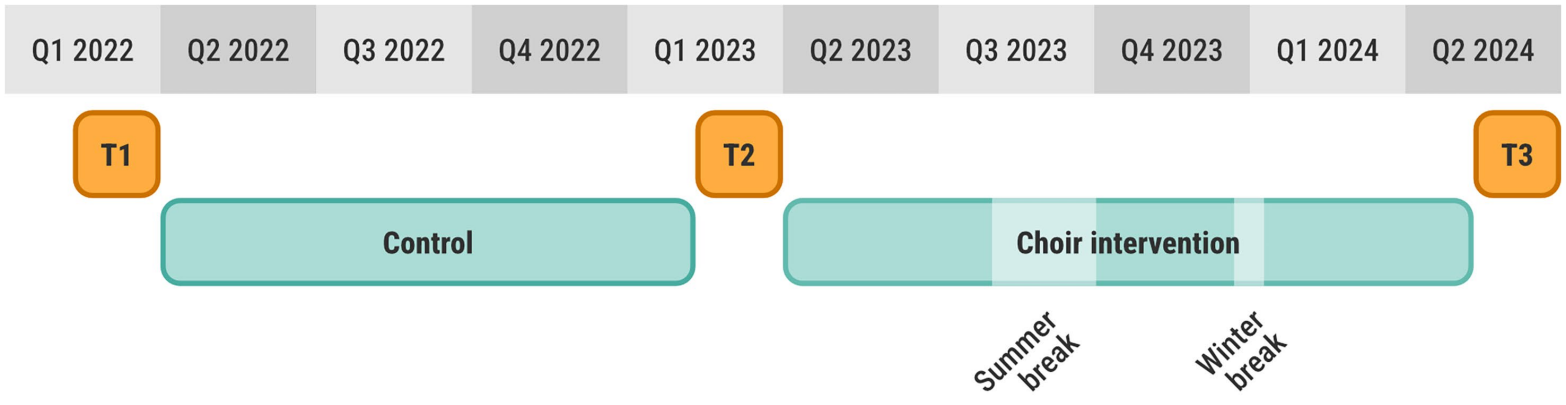
Study timeline showing the three data collections T1, T2, T3, as well as the control and choir intervention including breaks.

The choir intervention had a within-subject design, where each participant was measured at 3 different time points (T1-T3), approximately 11 months apart (see Figure 2). The first interval between T1 and T2 was a control period without any intervention or particular instructions for the participants. The choir singing intervention was carried out in the second interval between T2 and T3. Each data collection consisted of two (non-consecutive) days of testing (one day for behavioral tests and one day for MRI). In addition, a digital survey was filled out at home. Each data collection period lasted approximately two months and was sufficient to complete assessments for the full participant cohort.

The choir was assembled for the purpose of this study and consisted only of the study participants. The intervention included 47 weekly rehearsals, each with a duration of 90 min. There were scheduled breaks for summer and public holidays (see Figure 2). The choir was provided with practice material, such as sheet music and audio files, and instructed to also practice at home. The choir had 2–3 performances in front of an audience, as well as a few, small social events.

### 2.3 Instruments

#### 2.3.1 Psychological tests

The complete Wechsler Memory Scale (Wechsler, 1997) was administered at T1, T2 and T3. For the purpose of this study however, we focused on the episodic logical memory scales I (WMS-LMI) and II (WMS-LMII). The WMS-LMI assesses initial encoding and immediate memory span for structured verbal material. The WMS-LMII measures delayed recall of the same verbal material to assess retention, storage, and retrieval over time. We used the raw score instead of the scaled score, which is an age-corrected measure of the raw core in order not to correct for age twice.

General cognitive ability was measured as a control variable using the Raven’s 2 Progressive Matrices, clinical edition (Raven, 2018).

Mini-Mental State Examination (MMSE) (Folstein et al., 1975) is a cognitive measure with 20 items, with a maximum test score of 30. The test involves for example items regarding spatial and time orientation, such as what year and day it is. For this study, it was used as an inclusion criterion, with the cut-off set to below 26.

Geriatric Depression Scale, GDS, is a 20-item instrument, where a score above 6 might indicate depression. Participants who scored 11 or higher were excluded.

#### 2.3.2 fMRI experimental task

**Figure 3:**
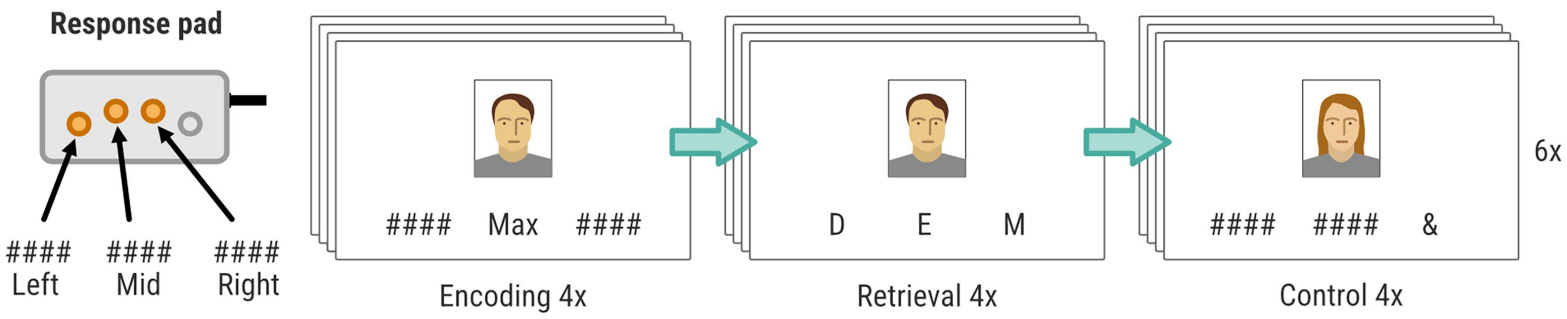
FN-PA task consisting of three blocks: encoding, retrieval, and control. Each block included four stimuli, and the procedure was repeated 6 times for each data collection. The position of the buttons on the response pad (left, mid, or right) was used by the participants to (1) confirm the name during encoding, (2) choose the correct first letter during retrieval, and (3) choosing the “&” sign during control. Only three of four available buttons on the response pad was used in the experiment.

Brain activity during episodic encoding and retrieval memory was measured with fMRI during a face-name paired associate learning (FN-PA) task (see Figure 3). We chose a task that has been used previously in aging research to allow for comparisons with previous work (e.g., Salami et al., 2012). The face stimuli were retrieved from the FACES database (Ebner et al., 2010), which contains naturalistic faces of young, middle-aged, and older individuals. We chose images illustrating neutral facial expressions, and an overall balanced distribution of gender and ethnicity. Below each face stimuli were three vertically aligned positions. The name that should be paired with the face was displayed in one of these positions and the others held strings of hash signs (“####”). The names were retrieved from lists of common Swedish names during the years 1960–1980. The positions of the character stimuli were pseudorandomized on each trial. The FN-PA task consisted of 3 blocks of encoding, retrieval, and control, which were repeated in that specific order 6 times. The visual stimuli were presented on a monitor behind the MR scanner viewed by the participants through a periscope mirror on the head coil. Importantly, all faces and names were replaced between T1, T2, and T3, in order to mitigate potential learning effects. As an additional measure to remove order effects, the face-name pairs were counterbalanced between T2 and T3, meaning that half of the participants, through pseudo-randomization, were presented with one set of face-name pairs during T2, and another set at T3. The remaining half were presented with the same sets but in reverse order. The task difficulty was such that participants should be able to correctly complete the task with few or no errors yet using some mental effort, in order to remain engaged during the whole task. We did not want brain activity to be confounded by participants failing the task or large task-specific training effects between time points. Rather, we wanted to characterize and compare brain activity related to mostly successful encoding and retrieval before and after the intervention. For this reason, no improvements nor deterioration on the behavioral data of the FN-PA task was expected, however a repeated measures ANOVA will be performed in order to confirm this assumption.

#### 2.3.3 Attendance

Presence at each choir practice session was recorded by the participants themselves, who filled out an attendance form.

### 2.4 Procedure

#### 2.4.1 Behavioral testing

The WMS-LMI-II was assessed by two licensed clinical psychologists at the Behavioral Lab of the Department of Psychology, Stockholm University. The procedure and test material was identical at all three timepoints.

#### 2.4.2 MRI

Before the participants entered the scanner, they received instructions on the experimental task, and performed a brief practice session of the FN-PA, with one block of encoding, retrieval, and control. The practice session used unique faces and names. These were however not replaced between T1, T2, and T3, in contrast to the face-name stimuli used in the actual experiment.

Each encoding block began with a written instruction displayed for 4 s (“Remember the person’s name and click on the button corresponding to that name”). Subsequently, there were 4 trials of face-name encoding interleaved with a pseudorandomized interstimulus interval (1.5/2.5/3/4.5 s). Each face-name stimulus was presented for 4 s during which participants had to encode the face-name pair and press the button corresponding to the position of the name on a standard response pad (vertically aligned buttons), using the index, middle, or ring finger.

The retrieval blocks also began with a written instruction displayed for 4 s (“Click the button that corresponds to the first letter of the correct name.”). Then came 4 trials of face-name retrieval, also interleaved a pseudorandomized interstimulus interval (1.5/2.5/3/4.5 s). Each visual stimulus was presented for 4 s and was composed of one of the faces encountered in the previous encoding block, as well as 3 letters distributed across the 3 positions below the face stimuli. One of these letters matched the first letter of the correct name. Thus, the participants had 4 s to indicate the correct name, using the index, middle, or ring finger, and the response box.

The control blocks similarly began with a written instruction displayed for 4 s (“Click the button that corresponds to &”). After the instructions there were again 4 trials of visual stimuli interleaved by a pseudorandomized interstimulus interval (1.5/2.5/3/4.5 s). Each visual stimulus, which was displayed for 4 s, was composed of a face, and distributed across the three positions below the face were an AND sign (“&”) and two hash signs (“#”).

Thus, there was no relation between the face and the symbols presented below and the participants simply had to press the button on the response box that corresponded to the “&” sign, using the index, middle, or ring finger.

### 2.5 MRI scanning parameters

The MRI data was acquired using a 3.0 T scanner (Siemens Magnetom Prisma, Siemens Healthcare GmbH) with a 20-channel head coil. All scans were performed at Stockholm University Brain Imaging Center (SUBIC, Stockholm, Sweden). Functional MRI was performed using a gradient echo pulse, echo-planar (EPI) T2*-weighted sequence with blood oxygenation level-dependent (BOLD) contrasts. The following parameters were used: repetition time (TR) = 2160 ms; echo time (TE) = 30 ms, field of view (FOV) = 20 cm; voxel size = 2 × 2 × 2 mm^3^; slice thickness 2,0 mm; flip angle = 70°. Whole brain image volumes were constructed from 68 contiguous axial slices in an interleaved slice order. Three “dummy” image volumes were acquired at the beginning of each session, to allow for T1 equilibration effects, but not saved. A high-resolution, three-dimensional spoiled gradient echo T1-weighted anatomical image was acquired in axial slice orientation: TR = 2530 ms; TE = 1.69 ms; inversion time (T1) = 1100 ms; FOV = 25.6 cm; voxel size 1.0 × 1.0 × 1.0 mm^3^; flip angle = 7°. A T2-weighted Fluid-Attenuated-Inversion-Recovery image was also acquired: TR = 5000 ms; TE = 388 ms; inversion time (T1) = 1800 ms; FOV = 25.6 cm; voxel size 1.0 × 1.0 × 1.0 mm^3^; flip angle = 120°.

### 2.6 Preprocessing of MRI data

The T1-weighted images were segmented using the longitudinal processing stream of the Computational Anatomy Toolbox (CAT12) version 12.8.2 (Gaser et al., 2024); the fMRI data were then preprocessed using SPM12 (Wellcome Department of Imaging Neuroscience, London, UK). Both software packages were implemented in MATLAB R2022b (MathWorks, Inc.).

For each participant and time points, the fMRI images were slice time corrected, realigned to the first image of the first session (Friston et al., 1995) and unwarped to remove residual variance caused by movement (Andersson et al., 2001). Thereafter, the unwarped images were coregistered to the average T1-weighted image (Ashburner and Friston, 1997) and finally normalized to MNI-space. Notably, the normalization was done using the average deformation field created during the CAT12 segmentation and were thus not biased by timepoint.

In addition, the CONN toolbox (Whitfield-Gabrieli et al., 2009) was used on the coregistered images to detect motion artifacts (intermediate settings; global-signal z-value threshold of 5; motion threshold of 0.9 mm).

### 2.7 Data analysis

#### 2.7.1 Psychological tests

A repeated measures ANOVA was conducted on each of the episodic memory tests WMS-LMI and WMS-LMII, to estimate the effect of time point. Tukey’s HSD was performed for pairwise comparison and controlling for type I errors. We had a directional hypothesis, expecting positive correlations on attendance and memory test scores, based on prior work on engagement dose effects. One-tailed tests and 95% confidence intervals were reported for these correlations.

#### 2.7.2 Functional MRI—comparing experimental conditions

The fMRI data were modeled at the first level for each time point separately using a general linear model (GLM) and the standard hemodynamic response function (HRF). The model included 7 regressors: (1) Encoding, (2) Retrieval, (3) Control, (4) Button presses, (5) Encoding instructions, (6) Retrieval instructions, (7) Control instructions. The button presses were included to control for motor output if participants should make mistakes. By default, the number of button presses were identical in the active conditions. Rest was part of the implicit baseline. The high-pass filter was set to 190 s. The contrasts of interest were Encoding−Control and Retrieval−Control. The resulting contrast images were smoothed using a Gaussian kernel with a full-width-at-half-maximum (FWHM) of 8 mm.

A second-level repeated measures analysis was performed on the contrast images from the first-level analyses, to compare Encoding/Retrieval between time points (using the flexible factorial option in SPM12). The resulting statistical parametric maps where corrected for multiple comparisons using Threshold-Free Cluster Enhancement (TFCE; Smith and Nichols, 2009), and a voxel-level family-wise error rate (FWE) of *p* < 0.05. The locations of significant clusters were cross-checked against the AAL3 atlas (Rolls et al., 2020) and the Harvard-Oxford atlas (Desikan et al., 2006).

#### 2.7.3 Functional MRI and attendance

To test an *a priori* directional hypothesis that rehearsal attendance is positively associated with hippocampal encoding activity, we ran voxelwise multiple regression within left and right hippocampal anatomical masks (AAL3), with attendance (T2–T3 interval) as the regressor of interest and age, sex, and Raven’s as covariates. Analyses were conducted in SPM12 on the T3–T2 Encoding–Control contrast images. Considering the small ROI sizes, we report parametric FWE-corrected results to avoid potential TFCE instabilities in very small search volumes.

#### 2.7.4 Analysis of task-dependent functional connectivity

A generalized psychophysiological interaction analysis (gPPI; McLaren et al., 2012) was performed to study the change in task-dependent functional connectivity between time points for Encoding–Control and Retrieval–Control using the CONN toolbox (22.v2407) (Whitfield-Gabrieli and Nieto-Castanon, 2012).

Prior to analysis, the preprocessed functional data were additionally denoised to remove potential confounding effects, including white matter and CSF timeseries, SPM covariates (realignment parameters and artifact regressors), session and task effects and their first order derivatives. Linear detrending and temporal band-pass filtering (0.008–0.09 Hz) were also applied.

The generalized psychophysiological interaction (gPPI) model was specified at the first level for each subject using the CONN toolbox. Task-relevant regions of interest (ROIs) were selected from the default atlases available within CONN. From the network atlas, we selected the (bilateral) lateral PFC (executive control), inferior frontal gyrus (strategic retrieval); from the anatomical atlas we selected the (bilateral) hippocampus (memory), posterior portion of the temporal fusiform cortex (face processing), anterior portions of the superior, middle, and inferior frontal gyri, and temporal pole (name processing/auditory–visual integration). Based on results (see section 2.10), we additionally included the posterior cingulate cortex from the networks atlas.

For each ROI, a separate gPPI model was constructed comprising (1) the physiological regressor (ROI-specific BOLD time series), (2) the psychological regressors (task condition-specific regressors convolved with the canonical hemodynamic response function), and (3) the interaction regressors (product terms of the physiological and psychological regressors), capturing condition-specific (context-dependent) changes in functional connectivity. Data from the three time-points were modeled as sessions, with session-specific effects.

Second-level group analyses were performed on the Fisher-transformed semipartial correlation coefficients derived from the gPPI interaction terms. For each contrast of interest (i.e., [(Encoding_T3–Control_T3) – (Encoding_T2–Control_T2)] and the corresponding contrast for Retrieval), a ROI-to-ROI analysis was conducted. In this analysis, connectivity maps for each ROI were entered into a random-effects general linear model to assess condition– and time point–dependent functional connectivity patterns. This approach allowed for the identification of significant changes in ROI-to-ROI connectivity associated with task conditions over time. Statistical significance was assessed using a false discovery rate (FDR) corrected threshold of *p* < 0.05.

## 3 Results

### 3.1 Descriptive statistics

Table 1 presents the descriptive statistics for the variables of interest and the sample size for each measure.

**Table 1:** Descriptive statistics for age (years), attendance (fraction), WMS-LMI and WMS-LMII (scores), MMSE (score), cognitive ability (score), and FN-PA (accuracy) across time points, including corresponding sample sizes.

Measure	*N*	Mean T1 (SD)	Mean T2 (SD)	Mean T3 (SD)
Age	39	69.3 (3.2)	-	-
Attendance	39	-	-	0.80 (0.10)
WMS-LMI	39	40.7 (8.6)	43.8 (8.3)	46.5 (8.4)
WMS-LMII	39	27 (6.3)	29.9 (6.9)	32.8 (6.9)
MMSE	39	29.5 (0.7)	29.4 (0.9)	29.2 (1.2)
Raven’s	37	-	-	104 (13.4)
FN-PA	36	16.9 (3.1)	18.1 (2.8)	17.8 (2.8)

### 3.2 Attrition bias

To evaluate potential attrition bias (see Figure 1), we performed a two-sample *t*-test comparing participants who dropped out (and were therefore excluded from the analysis) with those who completed the study. The groups were compared on the following measures: WMS-LMI, WMS-LMII, MMSE, gender, and age. No significant group differences were found on any of these variables (see Supplementary Table S1), i.e., we did not find any attrition bias in the measures of interest. Cognitive ability was only measured at T3, and could therefore not be evaluated.

### 3.3 Improved performance on WMS logical memory I and II

A repeated measures ANOVA showed a significant main effect of timepoint on WMS-LMI (*F*_(2, 76)_ = 18.4, *p* < 0.001, *η^2^G* = 0.075). Post-hoc pairwise comparisons using Tukey’s HSD showed that the participants’ WMS-LMI scores increased significantly from T1 to T2 (*p* = 0.001) and from T2 to T3 (*p* = 0.018).

Similarly, the repeated measures ANOVA for WMS-LMII showed a significant main effect (*F*_(2, 76)_ = 28.8, *p* < 0.001, *η^2^G* = 0.116), and post-hoc tests revealing both an increase from T1 to T2 (*p* < 0.001) and from T2 to T3 (*p* < 0.001).

### 3.4 Relation between rehearsal attendance and episodic memory performance

Since the reported improvement in episodic memory could to some extent be influenced by task-specific learning effects between time-points, we decided to further explore the performance increase in relation to individual differences in the number of attended choir sessions during the intervention. We conducted partial Pearson product moment correlations (one-tailed; expecting positive correlations) between attendance and difference scores in the two memory tests T2 − T1 (control period), and T3 − T2 (intervention), while controlling for age, sex, and cognitive ability. Although attendance could not cause the improvement between T2 and T1, it was included in the analyses to eliminate the possibility that a confounding variable affected both score improvement and attendance. There was no correlation between attendance and the increase in memory performance from T1 to T2 (WMS-LMI, *r* = 0.07, 95% CI [−0.275, 0.399], *p* = 0.34, *n* = 37; WMS-LMII, *r* = −0.12, 95% CI [−0.436, 0232], *p* = 0.74, *n* = 37). There was however a significant relation between the increase in memory performance from T2 to T3 and attendance on both tests (WMS-LMI, *r* = 0.34, 95% CI [−0.003, 0.605], *p* = 0.026, *n* = 37; WMS-LMII, *r* = 0.37, 95% CI [0.041, 0.632], *p* = 0.015, *n* = 37). Although the 95% confidence interval includes zero for WMS-LMI, the one-sided hypothesis test indicates a statistically significant positive partial correlation, consistent with the directional hypothesis.

Consequently, the performance increases in both immediate and delayed episodic recall after the intervention correlated with rehearsal attendance, which was not the case after the control period.

### 3.5 Behavioral data analysis based on the FN-PA task during fMRI

Accuracy on the FN-PA retrieval block was high and did not differ significantly between time points (Table 1). The task was intentionally constructed to minimize performance variability and disengagement, in order to interpret fMRI effects under relatively stable behavioral performance. It was however discovered that a few participants occasionally forgot to press the response button, resulting in outliers for button presses at each timepoint, defined as scores falling outside 1.5 × IQR from the upper (Q3) or lower (Q1) quartile. The outliers were mainly found in the encoding condition; 5 at T1, 4 at T2, and 5 at T3. Therefore, the number of button presses was entered as a nuisance variable in the fMRI analyses, both in the first and second level analyses. Repeated measures ANOVA confirmed that reaction times (from picture to button press) did not differ between time points for any condition.

### 3.6 Functional imaging analysis of the FN-PA task

Table 2 shows the results from the second-level analysis of brain activity during the FN-PA task, comparing the contrast images for Encoding–Control and Retrieval−Control between time-points. There were both decreases and increases in activity after the control period, but only decreases in activity after the intervention.

**Table 2:** Significant increases and decreases in brain activity after the control period and intervention period (TFCE FWE-corrected *p* < 0.05).

*Condition*/region	Peak (*x*,*y*,*z*)			*kE*	*TFCE*
Decreases T1→T2, retrieval−control					
Middle occipital lobe	–30	−94	14	37,428	4,007
Precentral gyrus	−48	6	24	13,557	3,237
Anterior insula	−33	26	2	450	1,457
Precentral gyrus	44	−3	62	983	1,304
Increases T1→T2, retrieval−control					
Paracingulate gyrus	−4	45	3	2,399	1772
Superior medial frontal gyrus	−4	54	30	40	1,267
Superior frontal gyrus	18	50	34	10	1,262
Decreases T2→T3, encoding−control					
Postcentral gyrus	−50	−27	58	1,535	1836
Posterior cingulate gyrus	6	−18	45	130	1,619
Postcentral gyrus	−62	−18	42	47	1,605
Precentral gyrus	8	−26	60	37	1,601

Peak = voxel coordinates (MNI-space) of peak activity in the cluster, kE = cluster size, TFCE = critical threshold value to yield a FWE rate of 5%.

**Figure 4:**
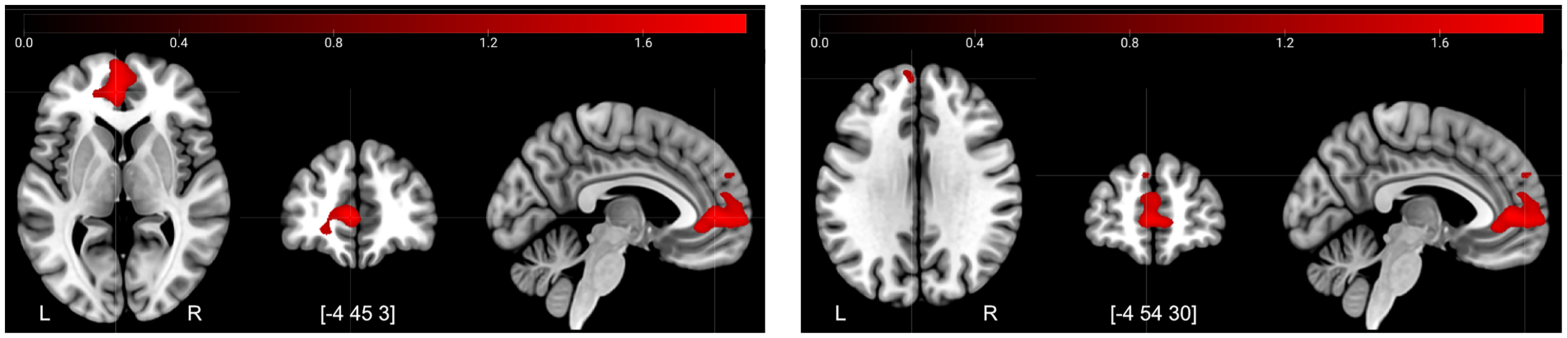
Decreases in activity at T2 compared to T1 for the contrast retrieval–control. Illustrated regions were significant at a TFCE FWE-corrected threshold of *p* < 0.05.

The decreases in activity after the control period for Retrieval–Control (Figure 4) corresponded mainly to a frontal-posterior cognitive control network (middle frontal gyrus, superior parietal lobe), visual association areas (cuneus/precuneus), motor areas (dorsal premotor region, presupplementary motor areas), and regions involved in memory retrieval—particularly during recognition tasks (anterior insula/posterior inferior frontal gyrus) (Buckner et al., 1996; Caviezel et al., 2020). In conjunction, these decreases suggest more efficient task-related information processing overall during the second session.

**Figure 5:**
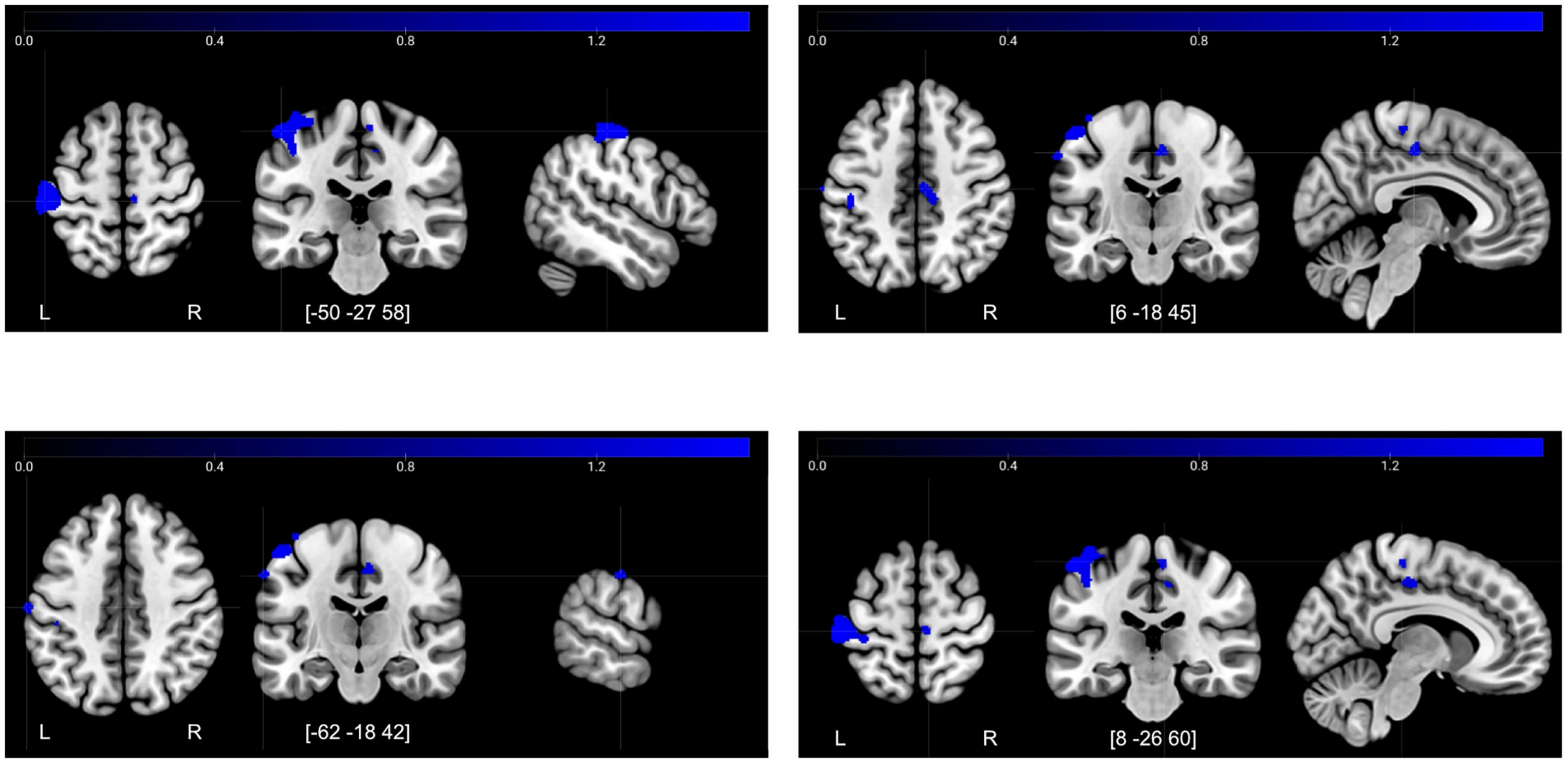
Increases in activity at T2 compared to T1 for the contrast retrieval–control. Illustrated regions significant at a TFCE FWE-corrected threshold of *p* < 0.05.

The increases in activity after the control period for Retrieval–Control (Figure 5), were found mainly in the pre-genual anterior cingulate cortex extending into the ventromedial PFC. In addition, there was a small cluster of increased activity in the dorsomedial PFC. These are areas associated with increased affective self-regulation (e.g., regulation of stress response) (Kerr et al., 2012).

**Figure 6:**
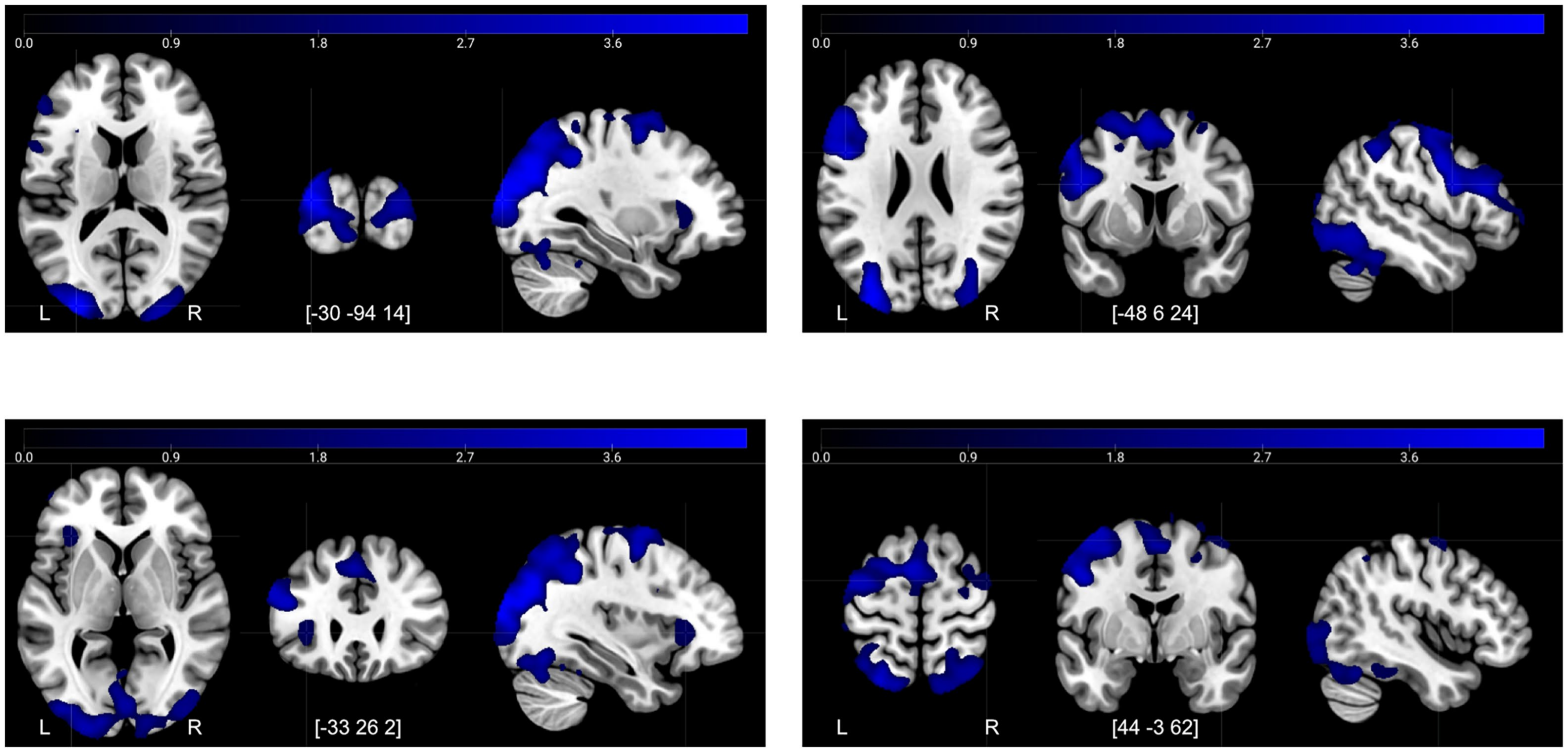
Decreases in activity at T3 compared to T2 for the contrast encoding–control. Illustrated regions significant at a TFCE FWE-corrected threshold of *p* < 0.05.

The decreases in activity after the intervention for Encoding–Control (Figure 6) were located in the sensory cortex—in the hand area and also in the face area. Interestingly, there was a cluster of decreased activity in the mid/posterior cingulate cortex, which is a region associated with attention allocation (Small et al., 2003) and response selection (Leech and Sharp, 2014). Based on this latter finding, we also included the posterior cingulate cortex as a ROI in the functional connectivity analysis.

#### 3.6.1 Relation between activity in the hippocampus and rehearsal attendance

Given the role of the hippocampus in encoding, and the observed behavioral increases in episodic recall associated with rehearsal attendance, we conducted an exploratory analysis to investigate whether rehearsal attendance would also be correlated with an increased activity in the left and/or right hippocampus after the intervention. This was implemented as a ROI-based voxelwise multiple regression in SPM12, restricted to anatomical hippocampal masks (derived from the AAL3 atlas) with FWE small-volume correction (attendance as regressor of interest; age, sex, and cognitive ability as covariates). Within the left hippocampus, attendance was positively associated with T3–T2 Encoding–Control activity (peak FWE, *p* = 0.010, *T*_(29)_ = 4.66, peak coordinate −36, −18, −21; cluster *k*_E_ = 52). Within the right hippocampus, a positive association was also observed (peak FWE, *p* = 0.032, *T*_(29)_ = 4.15, peak coordinate 39, −30, −10; cluster *k*_E_ = 17). We then performed a whole-brain analysis (same model) but there were no further findings.

In summary, people who attended more choir rehearsals had a greater increase from T2 to T3 in right hippocampal activity during encoding (vs. control) in the FN-PA task. An additional analysis of specificity confirmed that there were no similar relations between rehearsal attendance and difference scores in hippocampal activity T2–T1.

#### 3.6.2 Relation between brain activity and age

Exploratory analyses examined the relation between age and task-related brain activity across sessions (see Supplementary Tables S2-S4 and Figure S1 for full results). No significant associations were observed at T1. At T2, age was negatively associated with activity during both encoding and retrieval, whereas at T3 age was positively associated with activity in the right hippocampus and temporal regions.

#### 3.6.3 Functional connectivity

The gPPI analysis revealed two clusters of regions within which functional connectivity for Encoding–Control changed between T2 and T3. First, a cluster including the right PFC, the left posterior fusiform cortex, and the left hippocampus, within which connectivity increased (*F*_(2,34)_ = 6.0, *p* = 0.047); second, a cluster including the right lateral PFC and the left inferior frontal gyrus, within which the connectivity decreased from T2 to T3 (*F*_(2,34)_ = 5.38, *p* = 0.047). There were no changes in functional connectivity between T1 and T2.

## 4 Discussion

### 4.1 Intervention-related improvements in episodic memory

The current study examined episodic memory performance and related neural activity in older adults aged 65–75 years who participated in an 11-month choral singing intervention. Psychological testing and an fMRI-experiment were conducted at three time points; 11 months before the intervention (T1), just before the intervention (T2), and directly after the intervention (T3). This longitudinal design provided a robust framework for distinguishing the specific effects of the intervention from potential confounding factors. Such confounding factors may include general improvements in episodic memory due to repeated testing, increased familiarity with test procedures, or natural cognitive fluctuations over time. Without a longitudinal approach, these influences could be mistakenly interpreted as intervention effects. By measuring participants at multiple time points, the design helped isolate changes directly attributable to the intervention, thereby enhancing the validity of the finding. There were in fact significant improvements in episodic recall on WMS-LMI and WMS-LMII, as well as differences in brain activity during the FN-PA task, when comparing measurements before and after the passive control period. Importantly however, the improvements in episodic memory observed after the intervention scaled with the number of attended choir rehearsals. This was not the case for the improvements after the control period. The results align with those of Shinada et al. (2025). They found that participants who engaged in group music sessions showed significant improvement in WMS-LMII scores after the intervention, whereas the control group showed no significant improvement. In conjunction, these findings importantly strengthen the interpretation that choir singing had a positive influence on episodic memory. Because FN-PA accuracy was high and stable, we interpret intervention-related neural changes as reflecting more efficient encoding processes, consistent with the attendance-linked gains on standardized memory tests and hippocampal effects.

### 4.2 Intervention-related changes in brain activity during episodic memory performance

The fMRI analysis was aimed at comparing neural activity during episodic encoding and retrieval between the different time points. There were no differences in brain activity for Encoding–Control between T1 and T2, however a decrease in BOLD activity was found for Retrieval−Control in broad areas corresponding to networks involved in visual perception and visuomotor integration, planning and execution of motor output, and memory retrieval. Overall, these findings give a clear indication of more efficient neural processing in brain networks relevant for the task, which included perception and recognition of face/name stimuli, as well as selecting and executing targeted button-presses. It is also conceivable that participants were more relaxed and familiar with the procedure for the second scanning session, which may also have influenced brain activity. This interpretation is further supported by the simultaneously increased activity in the ventromedial and dorsomedial prefrontal cortices, which are areas associated with increased affective self-regulation (e.g., regulation of stress response) (Kerr et al., 2012). An upregulation of activity in these regions may be associated with a downregulation of stress-induced activity in other areas at T2. No further changes in brain activity were found for Retrieval–Control after the intervention at T3, which indicates that most task-specific improvements for this contrast occurred at T2, and the intervention had no observable effects on retrieval. Instead, we found decreased activity in four clusters after the intervention for Encoding–Control: in the hand and face areas of the postcentral gyrus/central sulcus, in the right supplementary motor area, and in the anterior portion of the posterior cingulate cortex (PCC). The first three clusters suggest less movement during task performance. The reduced activity in the face area could indicate that participants mirrored the different – albeit neutral – facial expressions in the stimuli more at T2 than T3. It is also conceivable that this finding is related to the intervention. Singing involves a high amount of training in body awareness and coordination; for example, regarding posture, breathing, and the voice apparatus (which includes training facial expressions) (Peultier-Celli et al., 2022). In addition, facial expressions are integral to singing, highlighting the training involved in coordinating facial movements with vocal performance (Livingstone et al., 2009).

The decreased activity in the PCC appears to be the more interesting finding. This region is an important part of the default mode network, which in general should be deactivated during performance of tasks requiring externally directed attention. The present findings are consistent with the conclusions of Natu et al. (2019), who posited that a decrease in PCC activity may prove advantageous during the encoding process. Rolls et al. (2023) found that the PCC was involved during encoding, particularly in the context of presenting multimodal stimuli such as face-name tasks, but that the PCC can simultaneously impede learning if over-activated. Maddock et al. (2001) highlighted connectivity between the PCC and hippocampus, suggesting that episodic memory encoding is facilitated by the PCC during autobiographical memory formation and the integration of contextual information. Here, the decrease in activity could therefore be indicative of enhanced efficiency of memory encoding, attributable to the intervention. Notably, there was no corresponding decrease of activity in the PCC after the control period.

#### 4.2.1 Relation between rehearsal attendance and activity increases in the hippocampus

Given the particular role of the hippocampus in memory encoding, and finding improvements in episodic memory after the intervention that scaled with rehearsal attendance, we performed an exploratory analysis to investigate whether rehearsal attendance would also be associated with activity in the hippocampus during encoding. Indeed, this is what we found. People who attended more choir rehearsals had a greater increase from T2 to T3 in hippocampal activity bilaterally during encoding in the FN-PA task, which was not observed from T1 to T2. Both the right and left hippocampus have been found relevant for face-name associations (Sperling et al., 2003), but it has also been shown that the left hippocampus is more involved in verbal memory while the right hippocampus is more involved in spatial and visual memory performance (Ezzati et al., 2016). In the FN-PA task, the names to be encoded with the faces could appear in three spatial locations, which may have contributed to the effects in the right hippocampus. Overall, the observed correlation between rehearsal attendance and both the behavioral improvements in episodic recall as well as the increases in hippocampal activity during episodic encoding, suggests a dose-effect relationship between choir singing and episodic memory performance.

#### 4.2.2 Intervention-related increases in hippocampal activity in older individuals

Although not central to our primary aims, exploratory analyses indicated that the relationship between age and task-related activity varied across sessions. Older individuals showed reduced activation at T2 but increased hippocampal and temporal recruitment at T3. Importantly, the systems recruited at T3 were directly relevant to the memory task (hippocampus, auditory and temporal–linguistic regions), which strengthens the interpretation that these effects reflect intervention-related modulation rather than non-specific changes. This pattern is consistent with models of age-related compensation (e.g., PASA) and may suggest that those further along in the aging process stand to benefit most from choir singing. Given the multimodal nature of choral participation, the recruitment of memory-relevant and auditory–linguistic circuits may help support episodic memory in older individuals. While exploratory and to be interpreted with caution, these findings raise the possibility that cultural interventions like choir singing could be especially valuable for maintaining cognitive health in later life.

#### 4.2.3 Intervention-related changes in task-dependent functional connectivity

The gPPI analysis showed a condition- and time-dependent increase in connectivity during Encoding–Control from T2 to T3 between the right lateral PFC, the left posterior fusiform cortex, and the left hippocampus. The right lateral PFC is involved in higher-order cognitive processes such as planning, inhibition, and decision making (reviewed in Miller and Cohen, 2001). The fusiform cortex is consistently engaged in face processing tasks (Kanwisher et al., 1997); left fusiform is typically less specialized than the right, but may contribute more to tasks that require visual processing of words alongside faces (Cohen and Dehaene, 2004; Tsapkini et al., 2011; Bi et al., 2014), which aligns well with the experimental task. The left hippocampus is a key region in memory encoding (Eichenbaum, 2001), particularly involving verbal and episodic memory encoding. Given the above reported findings, one might have expected the right hippocampus to have shown up in these results, however it would seem that when it comes to connectivity, the effect was stronger in the left hemisphere. It can be noted that many of the reported effects were to some extent bilateral, but did not reach significance in both hemispheres. Statistical power is therefore to be considered when discussing lateralization effects and we would not exclude bilateral findings from future hypotheses. Nevertheless, the combination of the hippocampus, fusiform, and lateral PFC illustrates a network of regions highly relevant to memory encoding in this task. Moreover, the condition- and time-dependent increase in effective connectivity in these regions further supports more efficient integration and information exchange between regions after the intervention, which was not observed after the control period. These findings support the idea that choir singing may enhance cognitive processes not only by improving isolated brain regions, but by strengthening network-level coordination relevant to memory and executive function.

Another significant cluster, involving again the right lateral PFC as well as the left inferior frontal gyrus, showed decreased task-dependent functional connectivity during Encoding–Control from T2 and T3. The left inferior frontal gyrus is primarily associated with cognitive control processes involved in memory retrieval (Badre and Wagner, 2007), rather than encoding. However, it may aid in the selection and organization of information also during encoding, particularly when strategic or demanding tasks necessitate controlled processing (Blumenfeld and Ranganath, 2007). Considering the observed direction of effect, it would thus seem that after the intervention, encoding required less cognitive control. Again, this is in line with a reversed PASA pattern.

#### 4.2.4 Choir singing and cognitive resilience

Although performance on the Face-Name Paired Associates (FN-PA) task did not differ significantly across time points, the observed neural changes suggest that choir singing may enhance the efficiency of memory-related processing rather than merely supporting compensatory mechanisms. This interpretation is supported by the apparent dose-dependent relationship between rehearsal attendance and both episodic memory improvement (WMS-LM) and right hippocampal activity during encoding. The multimodal nature of choir singing—engaging auditory, linguistic, motor, and social systems—may preferentially modulate encoding-related mechanisms, which are more resource-demanding and sensitive to lifestyle interventions (Rehfeld et al., 2017; Phillips, 2017). Enhanced functional connectivity between the hippocampus and fusiform gyrus further supports improved integration within memory-relevant networks. According to the PASA model, improved posterior processing may reduce reliance on frontal over-recruitment; the HAROLD and HERA models suggest a restoration of hemispheric specialization during encoding; and our findings according to the CRUNCH model supports more selective recruitment of task-relevant regions. Taken together, these findings indicate that choir singing may contribute to cognitive resilience in older adults by promoting more efficient and targeted neural engagement, even in the absence of measurable behavioral change.

### 4.3 Limitations and future directions

There is an inherent self-selection bias in the current study, as it was advertised as a choral singing study. It is not likely that individuals who do not enjoy singing would participate. This may limit how the results of this study generalizes to a broader population. We would however not advocate that *all* people engage in choir singing. Presumably, the usefulness and effectiveness of any cultural intervention will depend greatly on individual attitudes and preferences. However, since we do see interesting outcomes in this study, there is an added incentive to do further research both on choir singing and on other cultural activities. A societal goal would be to promote multiple evidence-based relevant, engaging, and accessible activities, that can be selected based on personal abilities and preferences. This study examined the effects of an 11-month choral singing intervention. Most previous music and choir-singing studies have used interventions of similar or even shorter duration, often ranging from a few weeks to several months, and these typically report small-to-moderate effect sizes [see for review Clark and Harding (2012) and Ito et al. (2022)]. However, a follow-up session would have clarified whether the observed effects were sustained over time. Future studies could examine whether longer engagement in regular singing would produce similar effects, and such studies would facilitate interpretation of potential public health policies and recommendations for health-promoting activities.

Several effects of time point could potentially be attributed to practice effects, due to repeated performance of the WMS tests and FN-PA task. It therefore strengthens the interpretation of an overall positive effect of the intervention, that choir singing may have a dose-effect relationship with both behavioral improvements and brain activity during episodic memory performance. Furthermore, many of the positive effects observed after the intervention, were not observed after the control period. An alternative design would be to include a passive or active control group. This would more effectively rule out the influence of practice effects and repeated testing. We acknowledge this as limitation of the study, however, we would like to promote more longitudinal studies cultural activities and aging, considering the benefits of this design; such as better control over individual differences, increased power, being able to observe individual trajectories, and avoiding cohort and period effects (Damoiseaux, 2017).

Our sample relatively narrow age range (65–75 years) may limit the ability to detect robust age-related differences and constrains the generalizability of our findings across the full spectrum of aging. This range was intentionally selected to focus on early older adulthood, a period when lifestyle interventions may be particularly effective for supporting cognitive resilience. Nonetheless, subtle age-related variability was still evident, as indicated by the correlation between age and right hippocampal activity during encoding. Future studies including a broader age distribution are needed to determine whether the effects of choir singing differ across later stages of aging and to better characterize age-related trajectories in neural and cognitive outcomes.

The data collected in this study does not reveal which aspect of choir singing that promotes episodic memory performance. There could be physical benefits, such as choir singing is a moderate physical activity involving controlled breathing, vocal effort, and coordinated posture, or increased socializing and making new friends, or cognitive training. The aim of the current study was more directed towards a proof of concept, and for the participants, it might not exactly matter which of a potential myriad of factors are at play. Nonetheless, it is of great interest to try to understand the specific mechanisms associated with the positive effect of choir singing, and the present study certainly encourages continued research.

It is also possible that the choir intervention at least partly could be associated with placebo effects, if the participants expect that the choral intervention will in some sense improve their performance (Colloca and Benedetti, 2005) because expectations and social contexts can lead to measurable changes in brain activity and performance. It could be argued however, for the very same reason, that attitudes matter and this placebo effect might actually correspond to very real and potentially lasting influences of for example attention and mood on memory performance. Such factors could potentially also interact with others mentioned, such as physical and social factors, to enhance the overall positive effect of choir singing on cognition.

Another notable limitation is the predominantly female sample, which may restrict the generalizability of the results. It is a known challenge within the choir community to recruit male choir members, which is essential to balancing the voices in mixed choirs (i.e., SATB: soprano, alto, tenor, bass). Despite the difficulty of generalizing the results to both female and male singers, we believe that the gender distribution of the recruited sample reflects that of choral singers in general, at least in the given context (Stockholm, Sweden).

Lastly, this study was not pre-registered. However, all exploratory analyses has been acknowledged as such in the reporting.

## 5 Conclusion

This study was preceded by a comprehensive preparatory study to develop a novel, carefully designed intervention aimed at ensuring a high quality an enjoyable experience (Napadow et al., 2024).

The present findings suggest that choral singing has a positive influence on episodic memory, particularly in older adult individuals who may have more to (re-)gain from increased physical, social, and cognitive activity. Taken together, the attendance-linked behavioral and hippocampal effects, along with task-dependent connectivity changes, are consistent with more efficient encoding mechanisms and align with models positing reduced reliance on compensatory frontal recruitment in aging. With an increasing aging population in many societies, it is becoming more and more important to find interventions that promote healthy aging, for instance by promoting the cognitive reserve. The findings in this study are in line with previous research suggesting that targeted interventions may benefit cognitive reserve in older adults (Håkansson et al., 2016; Kivipelto and Håkansson, 2017; Nyberg et al., 2020). This study provides further support for that cultural activities and choir practice in particular are interesting in this context, since they may mediate many positive factors in a format that is affordable, social, accessible, and enjoyable to most people.

## Data Availability

The datasets presented in this article are not readily available because the restrictions follows the regulations of the Swedish Ethical Review Authority. Requests to access the datasets should be directed to laszlo.harmat@lnu.se.
