# Supplementary material for: Singing for memory: neural and cognitive effects of a choral intervention in older adults
Source: Front Aging Neurosci. 2025 Nov 19;17:1679873. doi: 10.3389/fnagi.2025.1679873 (PMC12673369; doi:10.3389/fnagi.2025.1679873)
Supplement: Supplementary file 1 [file Table_1.DOCX]

# Supplementary materials

**Table S1. Negative relations between age and brain activity during Encoding-Control at T2.**

| Region |  | Peak (*x*,*y*,*z*) | | | *kE* | *TFCE* |
| --- | --- | --- | --- | --- | --- | --- |
| Cerebellum |  | –10 | –70 | –14 | 2067 | 1703 |
| Intracalcarine cortex |  | –20 | –69 | 8 | 918 | 1608 |
| Ventral diencephalon |  | 2 | –14 | –6 | 61 | 1523 |
| Cuneus |  | –4 | –90 | 30 | 315 | 1501 |
| Supracalcarine cortex |  | 24 | –63 | 15 | 80 | 1480 |
| Cerebellum |  | 9 | –56 | –16 | 14 | 1471 |
| Lingual gyrus |  | 14 | –69 | –4 | 8 | 1461 |

Peak = voxel coordinates (MNI-space) of peak activity in the cluster

*kE* = cluster size

*TFCE* = critical threshold value to yield a FWE rate of 5%

**Table S2. Negative relations between age and brain activity during Retrieval-Control at T2.**

| Region |  | Peak (*x*,*y*,*z*) | | | *kE* | *TFCE* |
| --- | --- | --- | --- | --- | --- | --- |
| Paracingulate gyrus |  | 9 | 26 | 36 | 85924 | 2765 |
| Inferior temporal gyrus |  | 54 | –56 | –24 | 1683 | 1889 |
| Lingual gyrus |  | –16 | –51 | –4 | 1752 | 1853 |
| Angular gyrus |  | 60 | –58 | 32 | 1266 | 1662 |
| Supramarginal gyrus |  | 45 | –38 | 54 | 1028 | 1618 |
| Superior parietal lobule |  | 24 | –51 | 69 | 240 | 1529 |
| Planum temporale |  | 56 | –33 | 14 | 16 | 1527 |
| Middle temporal gyrus |  | 62 | –60 | –6 | 346 | 1525 |
| Postcentral gyrus |  | 45 | -16 | 46 | 142 | 1520 |
| Inferior temporal gyrus |  | 45 | –66 | –8 | 8 | 1491 |

Peak = voxel coordinates (MNI-space) of peak activity in the cluster

*kE* = cluster size

*TFCE* = critical threshold value to yield a FWE rate of 5%

**Table S3. Independent samples t-test comparing completers versus non-completers at T1.**

| *Measure* | *Completers (n=38)* | *Non-completers (n=25)* | *t(df)* | *p-value* | *95% CI of Difference* | *Cohen’s d* |
| --- | --- | --- | --- | --- | --- | --- |
| Age | 69.3 (3.2) | 68.4 (2.5) | 1.16 | 0.252 | [-0.640, 2.391] | 0.30 |
| Gender | 0.1 (0.3) | 0.1 (0.3) | 0.13 | 0.895 | [-0.163, 0.186] | 0.03 |
| WMS-LMI | 40.9 (8.6) | 39.7 (8.8) | 0.54 | 0.590 | [-3.268, 5.698] | 0.14 |
| WMS-LMII | 27.2 (6.1) | 26.5 (6.2) | 0.48 | 0.635 | [-2.413, 3.926] | 0.12 |
| MMSE | 29.5 (0.6) | 29.4 (0.9) | 0.85 | 0.399 | [-0.225, 0.558] | 0.22 |

Excluded participants not included in analysis

Age = years at start of study

*df* = 61

**Table S4. Positive associations between age and brain activity during Encoding–Control at T3.**

| Region |  | Peak (*x*,*y*,*z*) | | | *kE* | *TFCE* |
| --- | --- | --- | --- | --- | --- | --- |
| Heschl’s gyrus |  | 51 | –12 | 6 | 2369 | 2324 |
| Planum polare |  | 44 | 2 | –22 | 2731 | 1935 |
| Planum temporale |  | –64 | –22 | 9 | 1547 | 1722 |

Peak = voxel coordinates (MNI-space) of peak activity in the cluster

*kE* = cluster size

*TFCE* = critical threshold value to yield a FWE rate of 5%

**Figure S1. Positive associations between age and brain activity during Encoding–Control at T3.** R = right hemisphere. The color gradient represents log *p*-values. The *z*-coordinate is presented for each slice.
